# Utilizing Edge-Oxidized Graphene Oxide to Enhance Cement Mortar’s Properties Containing Crumb Rubber: Toward Achieving Sustainable Materials

**DOI:** 10.3390/polym16142082

**Published:** 2024-07-21

**Authors:** Mohammed Alamri, Mohammad Khawaji

**Affiliations:** Department of Civil Engineering, College of Engineering, King Saud University, P.O. Box 800, Riyadh 11421, Saudi Arabia; mkhawaji@ksu.edu.sa

**Keywords:** strength, porosity, scanning electron microscopy, crumb rubber, sustainability

## Abstract

Scrap tires have become one of the most serious environmental issues worldwide in recent years. Exploiting this scrap has caught the attention of researchers in their efforts to conserve the environment. From a structural engineering materials perspective, a partial fine aggregate in cement mortar can be replaced by crumb rubber produced from scrap tires. This research mainly emphasizes the role of adding 0.1% edge-oxidized graphene oxide EOGO (by the weight of cement) in enhancing the properties of cement mortars containing 5%, 10%, and 15% of crumb rubber (by sand replacement). Cube and prism specimens were employed to investigate compressive and flexural strengths at 7- and 28-day curing ages. A porosity test was also conducted after 28 days of curing. In addition, a scanning electron microscopy (SEM) test was performed to investigate the effect of incorporating EOGO on the interfacial transition zone (ITZ). Results showed an enhancement of the mechanical properties of cement mortar, including compressive and flexural strengths, with the inclusion of EOGO in the mixes. The findings demonstrated that adding EOGO can improve the mechanical properties of mixes containing crumb rubber particles. Specifically, the mortar mix with 0.1% EOGO and 5% crumb rubber exhibited better performance compared with the virgin mix without rubber particles. Therefore, crumb rubber is viable for use as a sand replacement when EOGO is included.

## 1. Introduction

The increasing volume of tire waste is one of the major environmental issues at present [1,2]. By 2030, over five billion waste tires in the world are expected to be generated and stockpiled in landfills [3,4]. Scrap tires are flammable, and they are a main source of fires when stockpiled, especially during the summer [1,5]. The use of waste tires in the construction field can help find solutions to the issue of waste tire disposal [1]. This product of construction material is an environmentally friendly cement composite [1]. A potential solution for reusing scrap rubber related to construction materials is the use of wasted tire rubber as a replacement for coarse aggregate or sand in cement concrete mixtures.

Crumb rubber, which is produced from scrap tires, has been integrated into cement concrete mixtures in recent years [4,6,7]. Several studies have extensively investigated the properties of cement concrete by incorporating crumb rubber [3,8,9,10,11,12,13,14,15,16,17,18]. Previous studies showed that incorporating crumb rubber into cement concrete could enhance fatigue performance, post-crack strength, ductility, impact resistance, electrical resistivity, and toughness [8,9,10,11,12,13,14,15,16,17,18]. However, limited research has studied the properties of cement mortar containing crumb rubber as sand replacement [4,19,20,21,22]. A study reported that incorporating crumb rubber into cement mortar decreases the mortar’s compressive strength, flexural strength, density, and thermal conductivity [19]. In addition, the size of the rubber particles and the amount of added crumb rubber affect the mechanical properties of cement mortar. A previous study observed a significant reduction in compressive strength, flexural strength, drying shrinkage, and splitting tensile strength when the size of crumb rubber particles decreases and the volume of crumb rubber increases [20]. Not only are the mechanical properties affected by incorporating crumb rubber, but the internal structure of cement mortar is also affected, as reported previously [21]. There are studies that have shown that the porosity of cement mortar is correlated with the inclusion of crumb rubber in the volume replacing the sand [21,23]. Moreover, the study noticed that the slump flow and density of cement mortar containing crumb rubber are less than those of ordinary cement mortar, and the mortar’s flow and density are oppositely related to the crumb rubber particle size [21].

To improve the performance of cement composites, nanomaterials have been incorporated into cement concrete and mortar [4,24,25,26]. Recent advancements in nanotechnology have led to significant improvements in the properties of cement composites through the incorporation of nanomaterials such as carbon nanotubes (CNTs) [27]. Previous studies have observed notable improvements in the performance of cement composites. A previous study reported up to a 20% increase in compression resistance and up to a 34% increase in the modulus of rupture with the addition of 0.5% pristine and annealed multi-wall carbon nanotubes (MWCNTs) [28]. Another study has observed about a 25% increase in flexural strength with the addition of 0.08% weight of CNTs [29]. Furthermore, research by Cwirzen et al. found a 50% increase in compressive strength with CNT incorporation [30]. These studies’ findings highlight the potential of CNTs to significantly enhance the mechanical performance of cement composites.

Graphene oxide (GO) is one of the nanomaterials that can be added to cement composites to enhance their performance by promoting the cement hydration process [31]. Edge-oxidized graphene oxide (EOGO) is a specific type of carbon-based nanomaterial that is composed of carbon atoms bound together by C-C bonds [32,33]. Graphene oxide and edge-oxidized graphene oxide are chemically comparable; with the exception of EOGO, functionalized oxygen groups mainly exist only on the perimeters of the carbon sheets [34,35]. The effect of incorporating graphene oxide into cement paste has been observed in numerous studies [31,36,37,38]. A study conducted by Li et al. illustrated that EOGO contributes to the cement hydration process [39]. Additionally, Lv et al. reported that the cement hydration products containing graphene oxide form a distinct shape that aids in interlocking the cement hydration products [40]. This can lead to enhancements in the mechanical properties of the composite, as demonstrated by [41]. It is reported that graphene oxide contributes to and enhances cement characteristics in the early stages of the hydration process. Gong et al. (2015) showed that adding a very small amount of EOGO (less than 0.1% by weight of cement) could improve the compressive strength, tensile strength, and workability of the cement composite because it enhanced the process of cement hydration [31]. Graphene oxide has also been employed to improve cement composites containing recycled materials. A study revealed that adding GO to recycled aggregate-based cement composites notably enhances their mechanical and durability properties by improving the interfacial transition zones and increasing hydration reactions. Additionally, life cycle assessment studies suggest that recycled aggregate-based cement composites containing GO demonstrate superior environmental performance, particularly in terms of reducing CO_2_ emissions, compared to traditional cement composites [42].

Based on the literature, it can be concluded that crumb rubber has been utilized as a sand replacement material in cement concrete mixtures. This study investigates the effect of incorporating EOGO in cement mortar mixes containing various amounts of recycled crumb rubber, a feature that has not been previously explored. Since adding crumb rubber particles to cement mortar (as sand replacement) negatively affects the mechanical properties of cement mortar, the role of incorporating EDGO into mortar mixes containing crumb rubber particles is presented in this study.

## 2. Materials and Methods

In this section, materials, mix proportions, specimens’ preparation, and test methods are introduced as follows.

### 2.1. Materials

#### 2.1.1. Cement

**Figure 1:**
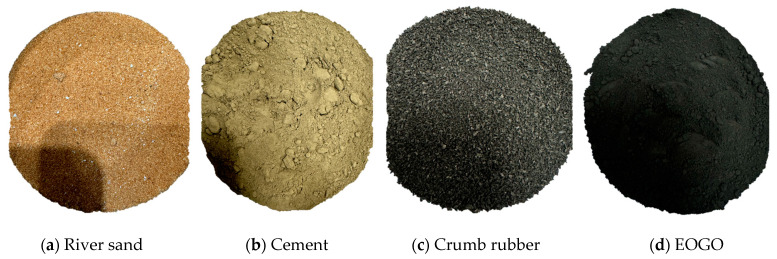
Materials used in this study.

Ordinary Portland Cement (OPC) Type I as shown in Figure 1a, which was supplied by Yamama Cement Company, Riyadh, Saudi Arabia, was used in the current study. OPC satisfies the requirements of ASTM C150 [43]. The specific gravity of cement is 3.15. The initial setting time is more than 45 min and the final setting time is less than 10 h. The chemical characteristics of OPC, as provided by the supplier, include 4% and 3% at most of MgO and SO_3_, respectively.

#### 2.1.2. Sand

River sand with an absorption of 0.5% and a specific gravity of 2.75 is utilized in this research as a natural sand to formulate mortar samples. The sand, shown in Figure 1b, was collected from an aggregate supplier located in Riyadh, Saudi Arabia. The river sand is well-dried in a draft oven at 100 ± 10 °C for 24 h to ensure that it is in a dry condition.

#### 2.1.3. Crumb Rubber

The fine-crumb rubber is used in this study as a replacement for natural sand. The material was provided by Ecofix Industrial & Trading Co. Ltd. (EITCO), Riyadh, Saudi Arabia. The crumb rubber particles were sieved in the laboratory, and particle sizes were found at a range of 0–1 mm. Figure 1c shows a photo of the crumb rubber batch used in this study.

#### 2.1.4. Edge-Oxidized Graphene Oxide (EOGO)

**Figure 2:**
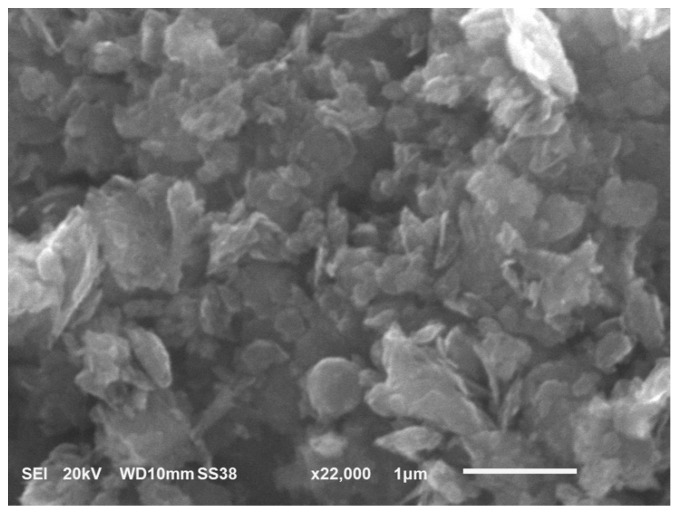
A sample of EOGO with 22,000× magnification.

Edge-oxidized graphene oxide (EOGO), shown in Figure 1d and used in this study, refers to graphene oxide nanoflakes that have customized oxygen groups on their edges. EOGO has been obtained from a supplier located in Orlando, Florida, in the United States. EOGO powder consists of 90% to 95% carbon, and the rest accounts for oxygen components, which represent about 5% to 10%. EOGO tiny particles have a nominal particle size of 500 nm and a surface area of 200–300 m^2^/g, as provided by the supplier specifications. Figure 2 shows the shape of the EOGO, taken by scanning electron microscopy (SEM).

### 2.2. Mix Proportions and Specimen Preparation

Eight mixes have been prepared in the laboratory to investigate the properties of cement mortar mixes containing 5%, 10%, and 15% of crumb rubber as sand replacements, and the effect of adding graphene oxide on these mixes. The water-to-cement (w/c) ratio is constant for all mixes at 0.48. Mixes are divided into two groups based on the presence of graphene oxide. Table 1 shows the mix proportions of all mixes in the current study.

Each cement mortar mix was cast with three replicates following ASTM C305 [44]. First, the dry sand and crumb rubber were placed in the mixer bowl and mixed slowly for three minutes. Then, Portland cement was added to the bowl and mixed with the sand and rubber batch properly for three minutes. After that, water was added slowly for a minute while mixing slowly for two minutes. Then, the mixer was switched to a high speed for 30 s. Finally, the mixer is switched to a slow speed for 30 s. For mixes containing graphene oxide, cement was mixed properly with graphene oxide in a dry condition before they were added to the sand and rubber in the bowl. Specimens were fabricated in cubes and prisms, and they were left for 24 h at room temperature before demolding. All specimens were then put in a water tank for curing until the testing date.

### 2.3. Test Methods

**Figure 3:**
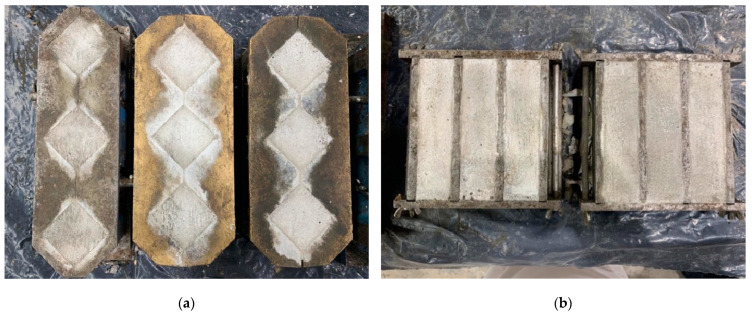
Cement mortar specimens in (**a**) cubic molds and (**b**) prism molds.

Compression and flexural tests were chosen to investigate the mechanical properties of cement mortar mixes in the current study. A porosity test has been performed on all mixes to confirm the performance results obtained from the mechanical tests. Cube and prism molds with 50 mm × 50 mm and 40 mm × 40 mm × 160 mm dimension sizes, respectively, were cast and cured in the testing program as shown in Figure 3a and Figure 3b, respectively.

#### 2.3.1. Compression Test

**Figure 4:**
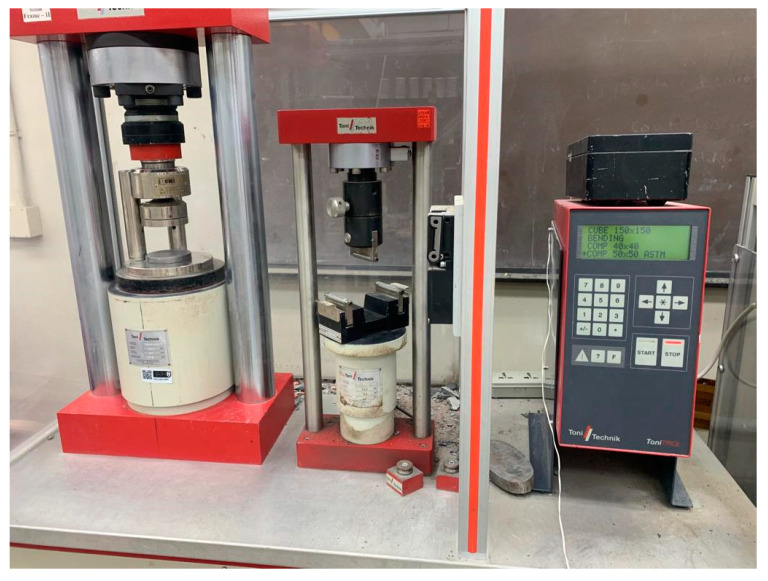
Universal testing machine.

Compressive strength was examined for different cement mortar mixes in accordance with ASTM C109 [45]. Cube molds were fabricated, cured, and tested to investigate the compressive strength of cement mixes after 7- and 28-day curing ages. A universal testing machine (Compression and Bending Test Plant ToniPRAX, Berlin, Germany) shown in Figure 4 was employed to measure the compressive strength. The rate of loading applied has remained constant at 1.5 kN/s throughout the test program.

#### 2.3.2. Flexural Test

To investigate the ability of cement mortar mixes to withstand bending loads, flexural strength was tested in the current study under ASTM C348 [46]. By using the universal testing machine shown in Figure 4, three-point loading was applied to specimens fabricated in prism molds after curing ages of 7 and 28 days. The rate of loading was kept constant throughout the test program at 0.05 MPa/s. The flexural strength of the specimen could be calculated by using Equation (1), where f is the flexural strength of the specimen in MPa, *P* is the ultimate applied load in *N*, and *L, b*, and *h* are the specimen length, width, and height, respectively, in mm.

(1) $$f=\frac{3PL}{2bh^{2}}$$

where

f= flexural strength, MPa;

P= the maximum applied load, N;

L= the sample length, mm;

b= the sample height, mm;

h= the sample width, mm.

#### 2.3.3. Porosity Test

To examine the internal structure of cement mortar mixes, a porosity test was conducted at a 28-day curing age following the guidelines outlined in ASTM C830 [47]. Initially, specimens’ weights were measured in water (*S*), then at saturated surface dry status (*W*), and finally at fully dried status (*D*). Porosity values were determined using Equation (2), where *P* represents the porosity of cement mortar mixes in percentage, and *W*, *D*, and *S* denote the sample weights in saturated surface-dried condition, oven-dried condition, and suspended in water, respectively, all measured in grams.

(2) $$P(\%)=\frac{W-D}{W-S}\times 100$$

#### 2.3.4. Scanning Electron Microscopy (SEM) Test

Samples of fractured specimens were taken directly after mechanical tests for microstructural investigation by using scanning electron microscopy (SEM). Samples are coated by putting them in a platinum substance. This step helps stop activation processes and assists in taking clear images for samples. Multiple touch panel scanning electron microscopy (JSM-6010PLUS/LA InTouchScope, Tokyo, Japan) is employed for SEM image purposes. After coating the samples, they are placed in a vibration-free sample chamber and vacuumed by a vacuum pump to avoid disturbance. After that, a beam of high-energy electrons with a very short wavelength is focused on the sample to detect the surface texture and chemical composition of the tested sample.

## 3. Results and Discussion

### 3.1. Compression Test

**Figure 5:**
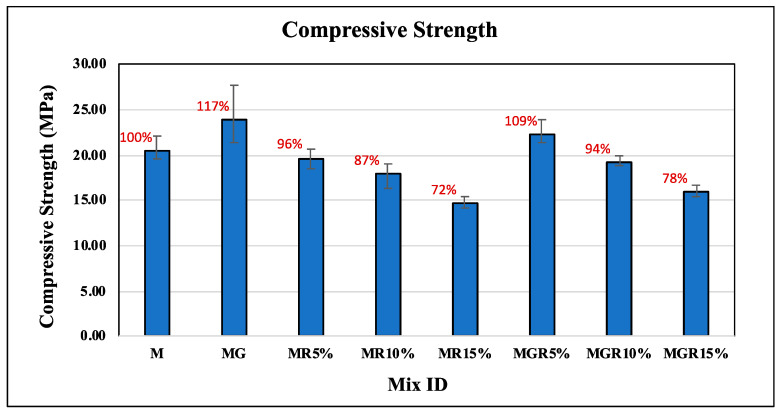
Compressive strength results of cement mortar mixes after 7 days of curing time.

**Figure 6:**
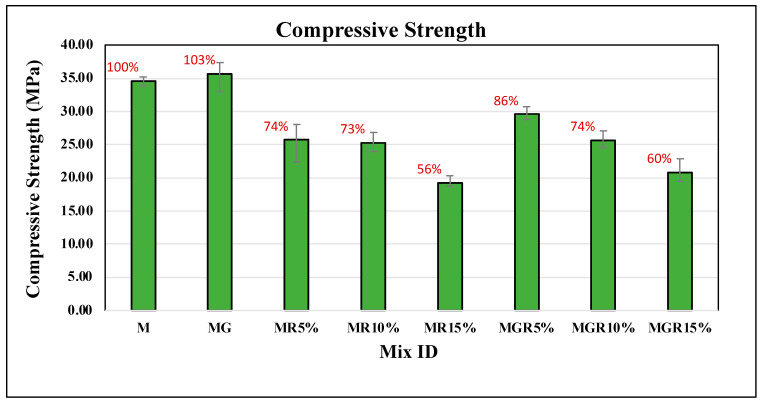
Compressive strength results of cement mortar mixes after 28 days of curing time.

Figure 5 and Figure 6 show the compressive strength results of cement mortar specimens for curing ages of 7 and 28 days with different amounts of crumb rubber replacement included, which are 0%, 5%, 10%, and 15%. In addition, the impact of incorporating 0.1% EOGO (by the weight of cement) is showcased within the figure as well as Table 2, aiming to observe its role in improving compressive strength. Each value represents the average of three repeated specimens. The percentages shown above bars in the figures represent the differences in compressive strength values compared to the control mix labeled “M”. It can be seen from the figure that there is a consistent reduction in compressive strength with the increase in crumb rubber amount in the cement mortar, aligning with previous studies’ findings as discussed in the introduction. This reduction is attributed to the hydrophobic nature of crumb rubber, which adversely influences the interfacial transition zone (ITZ) and weakens the bond between the cement composite and crumb rubber particles.

The role of incorporating 0.1% graphene oxide (EOGO) in mortars appears to improve the compressive strength of all mixes containing EOGO compared with ones without EOGO. The results reveal that incorporating 0.1% EOGO in the control mix leads to a notable improvement in compressive strength, with an average enhancement of 17% and 3% at 7 and 28 days of curing, respectively. This improvement is attributed to the role of EOGO in promoting cement hydration, thus enhancing overall compressive strength. Furthermore, for cement mortars containing 5% crumb rubber particles, the addition of EOGO resulted in a notable increase in compressive strength values by an average of 12% at 7 days and 13% at 28 days of curing compared to specimens without EOGO. Notably, even with the presence of 5% crumb rubber particles, incorporating 0.1% EOGO led to higher compressive strength performance than the control mixture, with an average improvement of 9% at 7 days of curing. For cement mortars containing 10% and 15% crumb rubber, the incorporation of EOGO enhances the compressive strength by an average of 7% at 7 days and 4% at 28 days of curing compared to specimens without EOGO.

The compressive strength results summarize the ability of EOGO to enhance the mechanical performance of cement mortars, particularly by mitigating the adverse effects of crumb rubber particle inclusion and promoting superior compressive strength characteristics.

### 3.2. Flexural Test 

**Figure 7:**
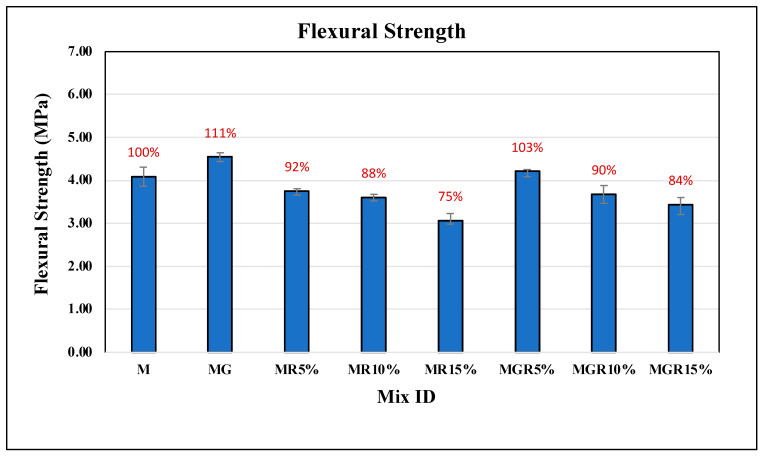
Flexural strength results of cement mortar mixes after 7 days of curing time.

**Figure 8:**
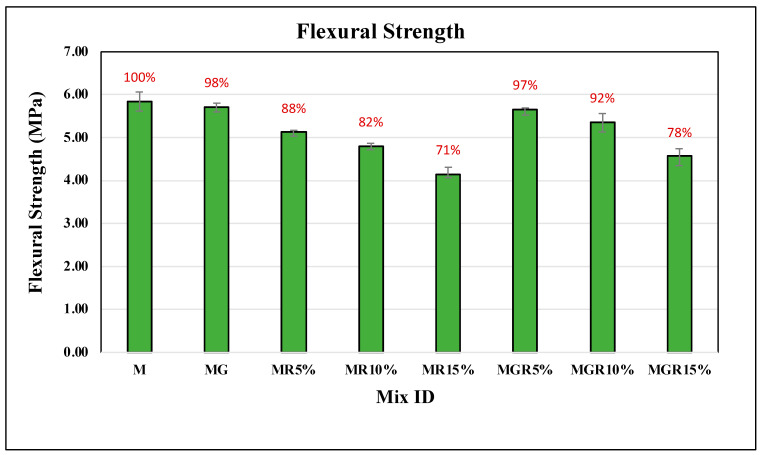
Flexural strength results of cement mortar mixes after 28 days of curing time.

The flexural test was conducted on all cement mortar mixes, with three replications for each mixture, at curing periods of 7 and 28 days. Figure 7 and Figure 8 present the flexural strength results for cement mortar containing different percentages of crumb rubber replacement (0%, 5%, 10%, and 15%). In addition, the flexural strength values for mixes containing EOGO were presented in Table 3 to facilitate a comparison of those mixes with mixes without EOGO. The findings indicate a slight decrease in flexural strength with increasing the percentage of crumb rubber in the cement mortars after either 7- or 28-day curing periods, which is consistent with existing literature as discussed in the introduction. This decrease is attributed to the hydrophobic nature of crumb rubber, which leads to higher air voids compared with natural sand. Therefore, replacing sand with rubber particles in a cement mortar mixture is expected to result in lowering the flexural strength due to increased air voids that allow affected stresses to transmit readily through the pores. For mixes containing 5% and 10% crumb rubber, the flexural test findings reveal a reduction in flexural strength by an average of 7% at 7 days and 14% at 28 days of curing. It is noticed that for 28 days of curing, there is a more pronounced reduction in flexural strength compared to the results for 7 days, owing to the improved degree of hydration facilitated by EOGO in the early stages of cement hydration. A further reduction in flexural strength is observed with the increase of crumb rubber by up to 15%.

Notably, the addition of EOGO showed a positive impact on flexural strength, as presented in Table 3. The results revealed that 0.1% graphene oxide could improve the flexural strength of the control mix by an average of 11% at the 7-day curing age, attributed to the filling properties of EOGO within the nanovoids of the mix. Additionally, regarding crumb rubber mortars, incorporating EOGO showed its potential to improve flexural strength in cement mortars containing up to 15% crumb rubber by an average of around 10% compared to rubber mortars lacking EOGO.

Overall, the flexural strength results suggest that incorporating EOGO has the potential to improve the mechanical performance of cement mortars, especially in counteracting the negative impacts of incorporating crumb rubber particles, so EOGO promotes improving flexural strength characteristics.

### 3.3. Porosity Test 

**Figure 9:**
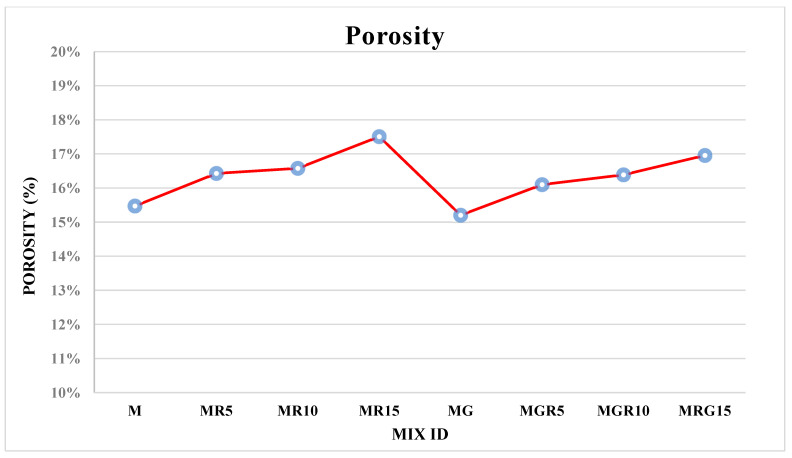
Porosity test results of cement mortars.

The porosity test was conducted on all mortar mixes after a 28-day curing age to examine the impact of adding EOGO on the porosity of cement mortar containing different amounts of crumb rubber particles. Figure 9 provides a summary of the porosity test results for all mixes. The findings indicate that the incorporation of EOGO resulted in a slight decrease in porosity, although not significantly so. This can be attributed to the presence of ultra-fine particles that filled the voids. The literature suggests that the increase in nanomaterials in cement mixes may contribute to a reduction in porosity [4]. Additionally, the findings indicate that incorporating crumb rubber leads to an increase in porosity as the rubber content rises from 0%, 10%, and 15% by 6%, 7%, and 13%, respectively, for mixes with no EOGO, and by 6%, 8%, and 12%, respectively, for mixes containing EOGO. These marginal increases stem from crumb rubber’s inclination to repel water and attract air, as noted by [48], resulting in the existence of voids or spaces in the interfacial transition zone (ITZ) and weakened bonds at the interface between the rubber particles and the cement mixes [49].

### 3.4. Scanning Electron Microscopy (SEM) Test

**Figure 10:**
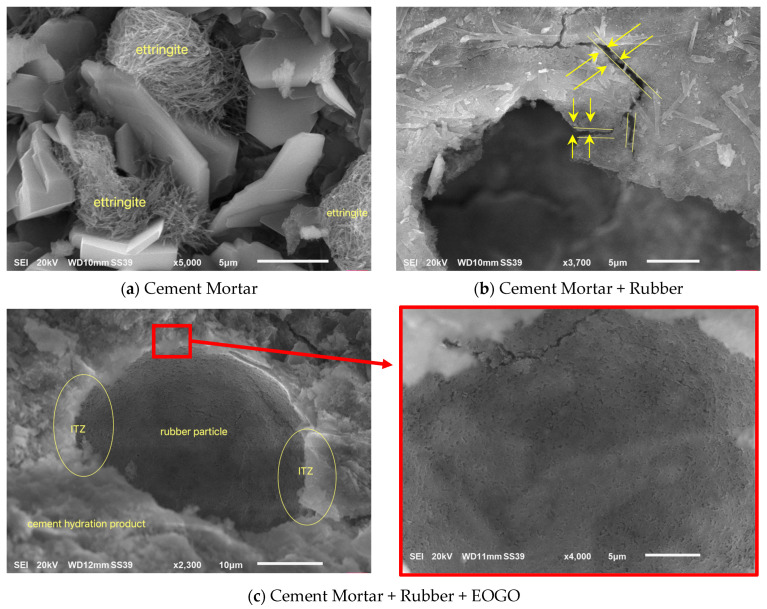
SEM images for different cement mortar mixes.

SEM images for different cement mortar mixes are shown in Figure 10. The products of cement hydration appeared in the form of C-S-H gel and CH plates (Figure 10a). Also, the formation of ettringite can be recognized in between the plates. Figure 10b shows a part of the crumb rubber particle and an adjacent area of cement mortar. As shown in Figure 10b, the presence of crumb rubber causes micro-sized cracks with a width of about 1 μm in the interface transition zone (ITZ). These cracks are initiated at ITZ and propagate to the cement mortar matrix as the externally applied load increases. This interprets the results of the mechanical strength obtained in this study. Adding EOGO to cement mortar containing crumb rubber contributes to promoting the cement hydration process around rubber particles, as shown in Figure 10c. The figure shows C-S-H gels surrounding the rubber particle. As a result, the ITZ is strengthened, and the micro-sized cracks are minimized. Moreover, EOGO is a hydrophilic material, which contrasts with the hydrophobic property of rubber, so EOGO helps attract water molecules to the surface of rubber particles. So, cement hydration products take place around rubber particles (Figure 10c). Adding EOGO to cement mortar containing crumb rubber assists in minimizing or preventing crack initiations in the ITZ areas. This feature explains the improvement of mechanical properties obtained in the current study.

## 4. Statistical Analysis

Laboratory test results were interpreted to analyze the performance between mixtures with and without EOGO regarding the mechanical properties. The statistical analysis involved comparing reference mixes (without EOGO) to those incorporating EOGO at varying crumb rubber contents of 5%, 10%, and 15%. A *t*-test was employed to determine if the mixes containing EOGO significantly differed from the reference mixes without EOGO. A 95% confidence interval, used in this study, is commonly used to evaluate the accuracy of the estimated statistics based on the variability of the data. The comparison focused on laboratory test results from the reference mix groups without EOGO (M, MR5%, MR10%, and MR15%), labeled as “Group #1”, and mixes that contain EOGO (MG, MGR5%, MGR10%, and MGR15%), labeled as “Group #2”.

Table 4, Table 5, Table 6 and Table 7 provide statistical data comparing mixes without EOGO to those containing varying amounts of crumb rubber and EOGO. The *p*-values indicate the significance of improvements in mixes with EOGO relative to reference mixes without EOGO. With a 95% confidence level for assessing test accuracy, *p*-values below 0.05 signify significant performance enhancements in the EOGO mixes compared to the reference mixes.

The *p*-values shown in Table 4, Table 5, Table 6 and Table 7 corroborate the laboratory test results, particularly concerning the early curing stage. An intriguing observation emerges regarding the mixes that contain EOGO. For mixes containing EOGO, the results indicate an improvement in mechanical properties compared to the reference mixes without EOGO. Specifically, the mixes with EOGO and 15% crumb rubber exhibit a significant performance enhancement over other percentages. This improvement can be attributed to EOGO’s role in filling the voids in the crumb rubber particles, thereby enhancing the mechanical properties. From Table 4, Table 5, Table 6 and Table 7, it is evident that the improvement in mechanical properties, in compressive and flexural strength, is more pronounced after 7 days in the early stage of curing compared to the 28-day curing period. This observation suggests that EOGO plays a significant role in enhancing the degree of hydration during the early stages of curing, as supported by the literature. 

In addition, an interesting finding emerges concerning the 5% crumb rubber mix containing 0.1% EOGO (MGR5%), based on *p*-value results. The MGR5% mix demonstrates better performance after 7 days of curing time compared to the virgin mix (M) without rubber particles and EOGO in terms of both compressive and flexural strength. Conversely, a slight reduction in performance is observed after 28 days of curing time.

Statistically, it can be concluded that incorporating EOGO enhances the properties of mixes containing crumb rubber particles compared with reference mixes without EOGO, particularly at 15% crumb rubber content. Moreover, EOGO mixes containing up to 5% crumb rubber exhibit the best behavior in resisting compression and flexural loadings compared to the virgin mix (M), with a confidence level of 95%.

## 5. Conclusions

This research explores the impact of integrating EOGO nanomaterial into cement mixes with varying crumb rubber contents. Cube and prism molds were utilized to assess the influence of different EOGO additions to crumb rubber mixes. The conclusions drawn from the experiment and analysis results are outlined as follows.

Substituting sand with crumb rubber particles resulted in a reduction in mechanical properties, such as compressive strength and flexural strength, when compared to the control mix.

The addition of 0.1% EOGO (by the weight of cement) resulted in improved mechanical performance of crumb rubber mixes at both 7- and 28-day curing ages.

Compared to the virgin control mix, the EOGO mortar mix with 5% crumb rubber exhibits better mechanical properties at a 7-day curing age. However, further increasing the crumb rubber content results in a reduction of these properties.

The porosity test results indicate that incorporating EOGO slightly decreases porosity due to ultra-fine particles filling voids, although the reduction is not significant. Conversely, increasing crumb rubber content from 0% to 15% leads to higher porosity.

SEM images reveal that cement hydration products form C-S-H gel and CH plates, with ettringite in between. The presence of crumb rubber induces micro-cracks at the ITZ, but adding EOGO enhances cement hydration around rubber particles, strengthening the ITZ and minimizing micro-cracks.

In future research, investigating the long-term durability of EOGO-enhanced crumb rubber cement mortars will be carried out. In addition, optimizing concrete mixes containing different percentages of EOGO and higher contents of rubber is planned to be investigated. This future research will also address the sustainability aspect by conducting a life cycle assessment and a life cycle cost analysis.

## Figures and Tables

**Table 1:** Mix proportions for different mixes.

#	Mix ID	Cement (g)	Water (g)	Sand (g)	Rubber (g)	EOGO (g)	Total
1	M	2225.5	1098.8	6120.2	0	0	9444.5
3	MR5	2225.5	1099.9	5814.2	130.3	0	9269.9
4	MR10	2225.5	1101.0	5508.1	260.7	0	9095.3
5	MR15	2225.5	1102.1	5202.1	391.0	0	8920.7
2	MG	2225.5	1098.8	6120.2	0	2.2	9446.6
6	MGR5	2225.5	1099.9	5814.2	130.3	2.2	9272.1
7	MGR10	2225.5	1101.0	5508.1	260.7	2.2	9097.5
8	MGR15	2225.5	1102.1	5202.1	391.0	2.2	8922.9

**Table 2:** The effect of EOGO incorporation into cement mortars containing different amounts of rubber on compressive strength.

0.0% EOGO			0.1% EOGO		
Mix ID	7-Day (MPa)	28-Day (MPa)	Mix ID	7-Day (MPa)	28-Day (MPa)
M	20.45	34.60	MG	23.92	35.73
MR5	19.63	25.75	MGR5	22.20	29.60
MR10	17.89	25.32	MGR10	19.21	25.64
MR15	14.65	19.27	MGR15	15.87	20.84

**Table 3:** The effect of EOGO incorporation into cement mortars containing different amounts of rubber on flexural strength.

0.0% EOGO			0.1% EOGO		
Mix ID	7-Day (MPa)	28-Day (MPa)	Mix ID	7-Day (MPa)	28-Day (MPa)
M	4.09	5.85	MG	4.55	5.71
MR5	3.76	5.12	MGR5	4.21	5.66
MR10	3.61	4.80	MGR10	3.68	5.36
MR15	3.06	4.15	MGR15	3.44	4.57

**Table 4:** Statistical analysis for group #1 mixes (M) versus group #2 mixes (MG).

	Group #1 (M)			Group #2 (MG)			*p*-Value	Significant?
	Value (Mean)	Standard Deviation	Variance	Value (Mean)	Standard Deviation	Variance		
Compressive (7 days)	20.45	1.34	1.80	23.9	3.3111	10.9632	0.0957	No
Compressive (28 days)	34.60	0.71	0.50	35.7	2.3543	5.5429	0.2542	No
Flexural (7 days)	4.09	0.23	0.05	4.6	0.1070	0.0114	0.0254	Yes
Flexural (28 days)	5.85	0.19	0.04	5.71	0.2327	0.0542	0.2435	No

**Table 5:** Statistical analysis for group #1 mixes (MR5%) versus group #2 mixes (MGR5%).

	Group #1 (MR5%)			Group #2 (MGR5%)			*p*-Value	Significant?
	Value (Mean)	Standard Deviation	Variance	Value (Mean)	Standard Deviation	Variance		
Compressive (7 days)	19.63	1.06	1.12	22.2	1.4908	2.2224	0.0356	Yes
Compressive (28 days)	25.75	2.99	8.92	29.6	1.0284	1.0576	0.0845	No
Flexural (7 days)	3.76	0.08	0.01	4.2	0.1196	0.0143	0.0058	Yes
Flexural (28 days)	5.12	0.24	0.06	5.66	0.3800	0.1444	0.0654	No

**Table 6:** Statistical analysis for group #1 mixes (MR10%) versus group #2 mixes (MGR10%).

	Group #1 (MR10%)			Group #2 (MGR10%)			*p*-Value	Significant?
	Value (Mean)	Standard Deviation	Variance	Value (Mean)	Standard Deviation	Variance		
Compressive (7 days)	17.89	1.47	2.15	19.2	0.5829	0.3397	0.1217	No
Compressive (28 days)	25.32	1.43	2.05	25.6	1.2875	1.6576	0.3938	No
Flexural (7 days)	3.61	0.08	0.01	3.7	0.2091	0.0437	0.3095	No
Flexural (28 days)	4.80	0.11	0.01	5.36	0.4593	0.2109	0.0885	No

**Table 7:** Statistical analysis for group #1 mixes (MR15%) versus group #2 mixes (MGR15%).

	Group #1 (MR15%)			Group #2 (MGR15%)			*p*-Value	Significant?
	Value (Mean)	Standard Deviation	Variance	Value (Mean)	Standard Deviation	Variance		
Compressive (7 days)	14.65	0.62	0.38	15.9	0.7047	0.4965	0.0444	Yes
Compressive (28 days)	19.27	0.88	0.77	20.8	1.7920	3.2112	0.1327	No
Flexural (7 days)	3.06	0.14	0.02	3.4	0.2051	0.0421	0.0296	Yes
Flexural (28 days)	4.15	0.21	0.05	4.57	0.0895	0.0080	0.0257	Yes

## Data Availability

No new data were created or analyzed in this study. Data sharing is not applicable to this article.
